# Increase of reactive oxygen species contributes to growth inhibition by fluconazole in *Cryptococcus neoformans*

**DOI:** 10.1186/s12866-019-1606-4

**Published:** 2019-11-06

**Authors:** Nadir Hani Dbouk, Madison Bailey Covington, Kenny Nguyen, Srikripa Chandrasekaran

**Affiliations:** Department of Biology, Furman University, Greenville, SC USA

**Keywords:** Antifungal treatment, Antioxidants, ROS, Metallothionein

## Abstract

**Background:**

*Cryptococcus neoformans*, a basidiomycetous yeast, is a fungal pathogen that can colonize the lungs of humans causing pneumonia and fungal meningitis in severely immunocompromised individuals. Recent studies have implied that the antifungal drug fluconazole (FLC) can induce oxidative stress in *C. neoformans* by increasing the production of reactive oxygen species (ROS), as presence of the antioxidant ascorbic acid (AA) could reverse the inhibitory effects of FLC on *C. neoformans*. However, in *Candida albicans*, AA has been shown to stimulate the expression of genes essential for ergosterol biosynthesis. Hence, the contribution of ROS in FLC-mediated growth inhibition remains unclear.

**Results:**

In order to determine whether counteracting ROS generated by FLC in *C. neoformans* can contribute to diminishing inhibitory effects of FLC, we tested three other antioxidants in addition to AA, namely, pyrrolidine dithiocarbamate (PDTC), retinoic acid (RA), and glutathione (GSH). Our data confirm that there is an increase in ROS in the presence of FLC in *C. neoformans*. Importantly, all four antioxidants reversed FLC-mediated growth inhibition of *C. neoformans* to various extents. We further verified the involvement of increased ROS in FLC-mediated growth inhibition by determining that ROS-scavenging proteins, metallothioneins (CMT1 and CMT2), contribute to growth recovery by PDTC and AA during treatment with FLC.

**Conclusion:**

Our study suggests that ROS contributes to FLC-mediated growth inhibition and points to a complex nature of antioxidant-mediated growth rescue in the presence of FLC.

## Background

Eukaryotic pathogens, including pathogenic fungi are an important cause of death in immunocompromised patients and can colonize immunocompetent individuals [1]. Cryptococcal meningitis caused by *Cryptococcus neoformans* is the leading cause of fungal central nervous system infection in the world, especially among persons suffering from HIV/AIDS [2, 3]*.* According to CDC reports, annually one million global cases of cryptococcal infections occur, accounting for up to 600,000 mortalities and about one-third of all AIDS-associated deaths. Despite the severity of cryptococcosis, unfortunately current treatments for cryptococcal infections are inadequate. A main barrier to establishment of an effective antifungal drug therapy is increased drug resistance in fungi [4–6].

Compared to other anti-cryptococcal drugs, fluconazole (FLC) is the more affordable and less toxic alternative, which is most commonly prescribed in geographic locations where cryptococcosis is most prevalent [7, 8]. FLC is the drug of choice for moderate pulmonary infections. For central nervous system infections, a combination of more expensive fungicidal drugs amphotericin B and flucytosine are administered [9, 10]; however, the combination of these two drugs produces more toxic side effects for the host.

A well-established mechanism of action of FLC is the inhibition of Erg11, which is one of the key enzymes participating in the synthesis of ergosterol, an important component of the plasma membrane [11]. One factor that contributes to failure of FLC-based therapy is the development of drug resistance. FLC resistance in *C. neoformans* occurs primarily via development of aneuploid cells with elevated levels of Erg11, which prevents diminishment of ergosterol [12]. Other causes for FLC resistance in pathogenic fungi include accumulation of mutations in *ERG11* [13] and via drug efflux pumps [14, 15]. Importantly, the mechanisms through which FLC leads to formation of aneuploid and FLC resistant cells remain largely uncharacterized.

While diminishment of ergosterol is a well-documented cause of FLC-mediated growth inhibition of *C. neoformans*, additional possible effects of FLC on *C. neoformans* cells have been proposed. FLC treatment has been shown to cause an increase in reactive oxygen species (ROS) in *Candida albicans* [16–18] and most recently in *C. neoformans* [19]. ROS are molecules with unpaired, highly reactive electrons called free radicals, generated during basic cellular processes, or due to external stress-inducing conditions, including environmental pollutants, foreign compounds such as drugs or chemicals, and exposure to X-rays [20]. Free radicals are highly reactive and unstable and excessive amounts of ROS are known to cause cell damage and trigger apoptosis. Generation of high amounts of free radicals can be harmful to biological macromolecules, as it can cause modification of DNA bases [21], lipid peroxidation, and protein carbonylation [22] leading to damage due to oxidative stress. Some examples of ROS include hydroxyl radicals, hydroxide anion radicals, singlet oxygen, hydrogen peroxide, hypochlorite, nitric oxide radicals, and peroxynitrite radicals. FLC-mediated increase in ROS could contribute towards oxidative stress in *C. neoformans.* Consistent with FLC-triggered ROS contributing to growth inhibition, co-treatment of *C. neoformans* cells with FLC and the antioxidant ascorbic acid (AA) was shown to partially rescue *C. neoformans* cells from FLC-mediated growth inhibition [19]. Similarly, co-treatment of *C. albicans* cells with the anti-fungal drug miconazole and a synthetic antioxidant, pyrrolidine dithiocarbamate (PDTC), has been shown to increase the Minimal Inhibitory Concentration (MIC) of miconazole [18]. These studies suggest an additional effect of anti-fungal azole drugs on pathogenic fungi, which is inducing oxidative stress via an increase in ROS content.

Interestingly, treatment of *C. albicans* with AA has been shown to increase expression of the gene *UPC2*, which is involved in regulating ergosterol biosynthesis [23, 24]. This finding suggests that AA might be functioning indirectly to regulate ergosterol levels, which is by counteracting FLC-mediated inhibition of ergosterol biosynthesis. Hence, whether ROS increase triggered by FLC contributes to growth inhibition elicited by FLC remains unclear.

The metal copper has been shown to be essential for virulence of *C. neoformans* [25]. Lack of a copper transporter, CTR4, led to reduced virulence in cryptococcosis models in mice [26]. During infection by *C. neoformans*, copper acquisition and increased copper levels is essential for melanin formation, which confers virulence to *C. neoformans* [27]. While elevated copper is essential during infection, increased copper can be toxic as it contributes to increased production of ROS, due to its participation in oxidation and reduction reactions [28]. To counteract harmful effects of copper, *C. neoformans* increases expression of metallothionein genes, *CMT1 and CMT2*, which bind to and sequester copper [29]. Previous studies have shown that *C. neoformans* mutants lacking metallothionein genes exhibit attenuated virulence [30] and show an increased sensitivity to FLC [19]. These findings suggest that Cmt1 and Cmt2 proteins allow for reversal of some of the harmful effects of ROS generated in the presence of FLC.

The purpose of this study was to perform a more rigorous test to determine if ROS plays a role in influencing sensitivity to FLC in *C. neoformans*. In order to determine whether it is the antioxidant properties of AA that caused rescue of *C. neoformans* growth inhibition, we tested three alternative known antioxidants for their ability to reverse the effects of FLC on the wild type as well as on metallothionein deficient mutants. Our data suggest that treatment with FLC leads to increase of ROS and this oxidative stress may further contribute to FLC-mediated growth inhibition. Furthermore, this study suggests that lowering ROS is not the only contributing factor to the antioxidant-mediated growth rescue and points to the complex nature of the physiological effects of FLC.

## Results

**Fig. 1:**
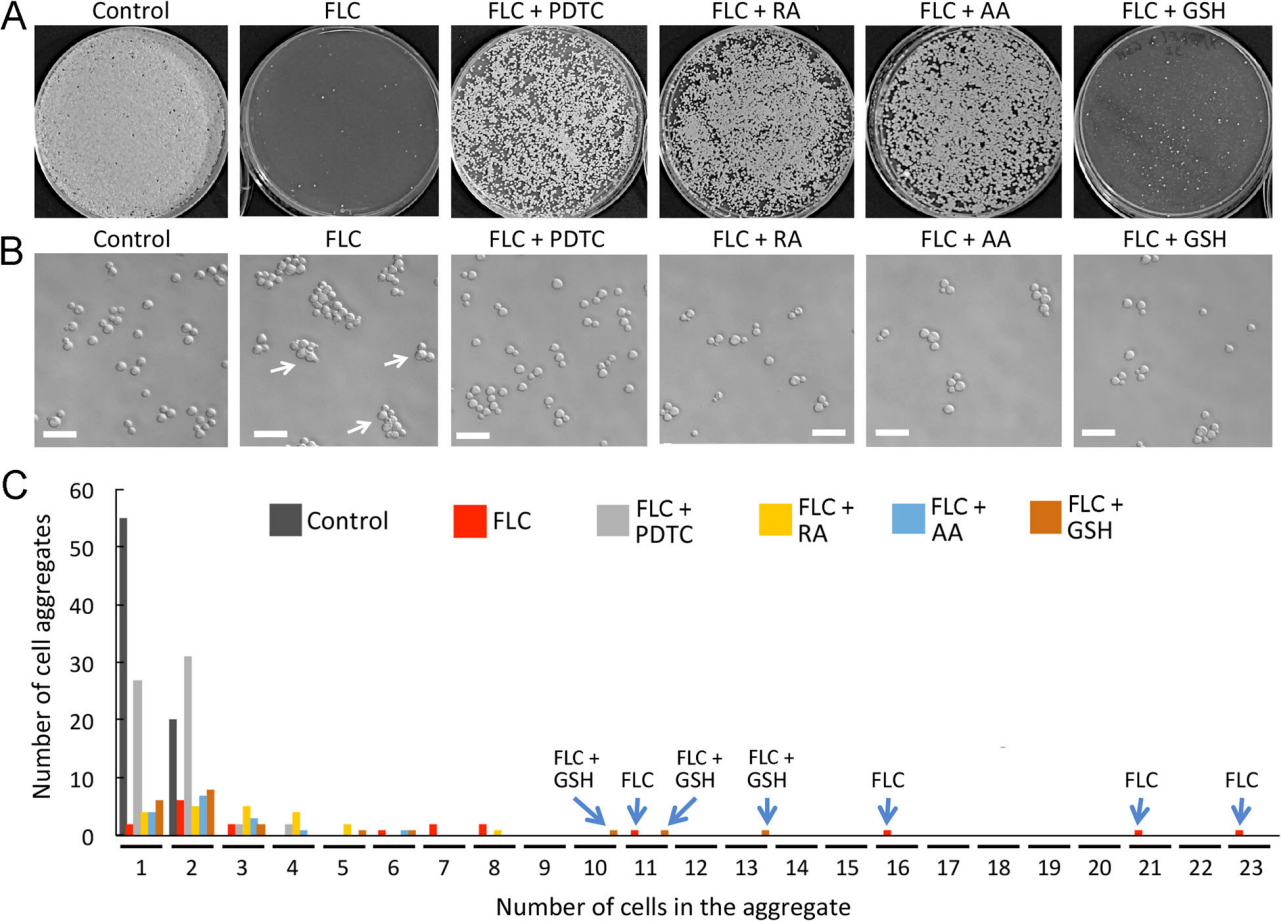
PDTC, RA, AA and GSH reverse growth inhibitory effects caused by FLC. **a** 10,000 cells of *Cryptococcus neoformans* wild type strain (H99) were spread on YPD semi-solid media (Control) or YPD media supplemented with FLC at 32 μg/mL alone or with addition of tested antioxidant compounds as indicated. Growth inhibition was observed in the presence of FLC and restored growth was observed to various degrees in the presence of antioxidants at 48 h after plating. The presence of Ascorbic Acid (AA) at 10 mM, Retinoic Acid (RA) at 1 mM, and pyrrolidine dithiocarbonate (PDTC) at 10 μM led to the greatest growth rescue while glutathione (GSH) at 10 mM showed marginalized recovery from FLC treatment. **b** Cells were treated with the chemicals as indicated in **a**, except in liquid cultures at room temperature. (25^0^ C) for 16 h. Cells treated with FLC at 32 μg/mL became clustered and multi-budded (depicted by arrows) indicative of compromised cytokinesis. Wild type morphology of cells incubated in the presence of FLC and the antioxidants suggests that the antioxidants rescued cells from cytokinesis defects caused by FLC. **c** Graph showing distribution of cell aggregates in population of cells treated as in **b**. Addition of an antioxidant (especially, PDTC, RA, and AA) reduced the number of cell aggregates. Bars indicate 20 μm

**Fig. 2:**
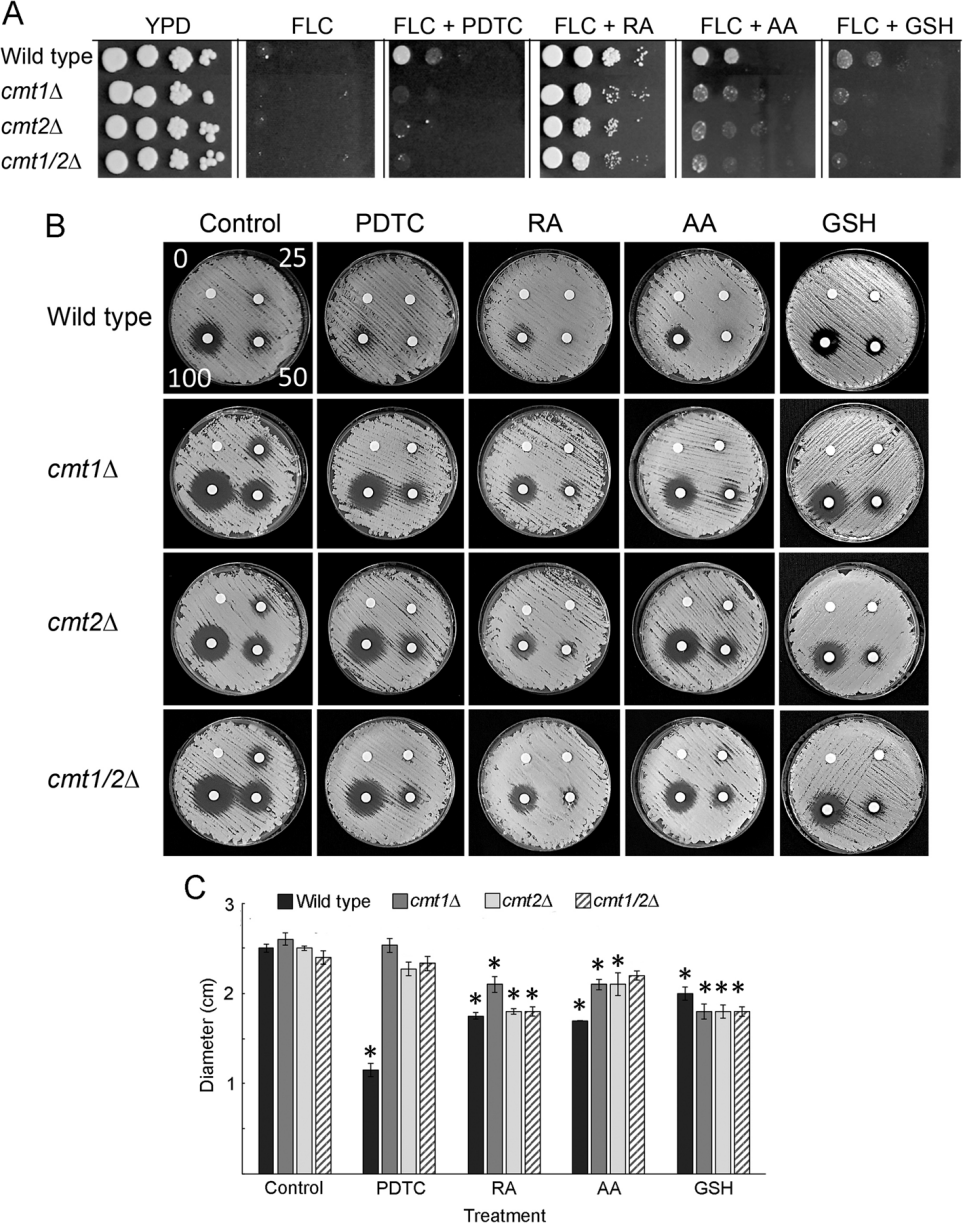
Metallothioneins contribute to antioxidant-mediated growth rescue in the presence of FLC. **a** Growth spot assay of wild type (H99), and the *cmt1*Δ, *cmt2*Δ, *cmt1/2*Δ mutants on YPD, or YPD supplemented with FLC (32 μg/mL) or FLC and an antioxidant (10 mM PDTC, 1 mM RA, 10 mM AA, 10 mM GSH). **b** 2 × 10^6^ of wild type cells (H99) or metallothionein mutants were spread on YPD semi-solid media or YPD media supplemented with antioxidants at concentrations as described in **a**. Discs containing increasing amounts of FLC (25, 50, or 100 μg) were placed on media and the growth inhibition zones were examined after 2 days of incubation at 25 °C. **c** Graph based on quantification of the results of the experiment described in **b** (based on three replicates). Error bars indicate standard deviation. Stars indicate significant growth rescue by the antioxidant as compared to the control treatment (*p* < 0.05)

**Fig. 3:**
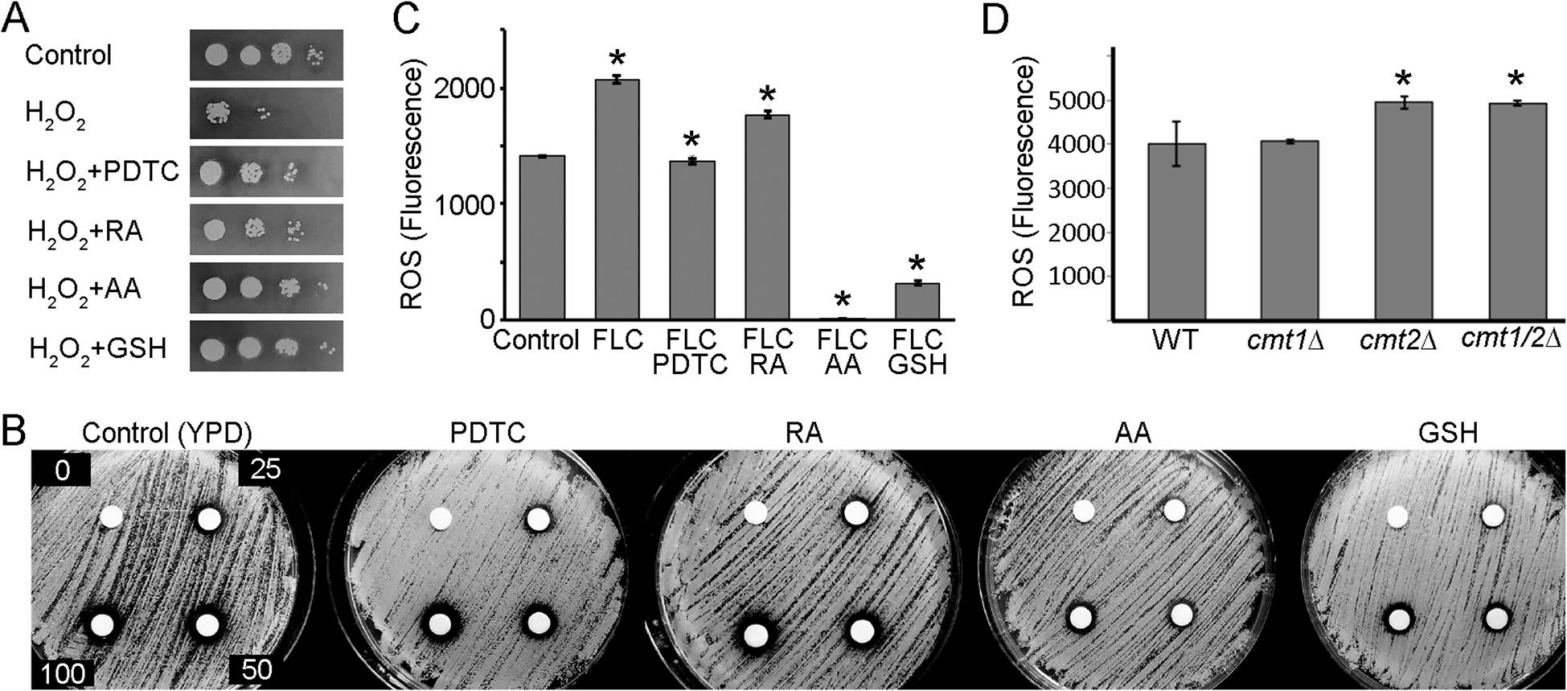
Analysis of the antioxidant potential of the tested compounds. **a** Growth spot assay showing wild type (H99) cells incubated on either YPD alone, YPD containing 3 mM of hydrogen peroxide (H_2_O_2_), or YPD containing 3 mM H_2_O_2_ and an antioxidant (10 μM PDTC, 1 mM RA, 10 mM AA, or 10 mM GSH). **b** Disc diffusion assay represents resistance of wild type strain (H99) to increasing concentrations of H_2_O_2_ (25, 50, 100 mM). ~ 2 × 10^6^ of cells were spread over YPD plates containing no antioxidant, or 10 μM PDTC, 1 mM RA, 10 mM AA, or 10 mM GSH. All antioxidants rescued growth in the presence of 3 mM H_2_O_2_ with AA and GSH having more visible effect as compared to PDTC and RA. **c** A fluorescence assay to measure ROS in wild type strain (H99) was performed, wherein greater fluorescence indicates higher levels of ROS. There is an increase of ROS in the presence of FLC (*p* < 0.01), and ROS is reduced in the presence of FLC and an antioxidant (*p* < 0.01, either 10 μM PDTC, 1 mM, RA, 10 mM AA, or 10 mM GSH) as indicated by a star. **d** Fluorescence assay to measure ROS in wild type (H99) and in metallothionein mutant strains (*cmt1*Δ, *cmt2*Δ, *cmt1/2*Δ) was performed where the cells were treated with 32 μg/mL FLC. A significance of the increase in ROS in the mutant as compared to the wild type control is indicated by a star, based on three replicates (*p* < 0.05)

We wished to determine whether antioxidants with diverse chemical structures and modes of action could alleviate FLC-mediated growth inhibition of *C. neoformans.* In addition to AA that has been previously shown to reduce growth inhibition in the presence of FLC in *C. neoformans* [19], we tested three chemically unrelated antioxidants: pyrrolidinedithiocarbamate (PDTC), retinoic acid (RA), and a reduced form of glutathione (GSH). The concentrations of AA, PDTC, and GSH were established based on previous studies [18, 19, 31]. The concentration of RA was established as the smallest concentration that rescued growth of *C. neoformans* cells in the presence of hydrogen peroxide (as later indicated in Fig. 3a). Growth of cells on plates with media supplemented with the respective amounts of the antioxidants and lacking FLC was not inhibited as compared to the control YPD media (as indicated in Fig. 2b). As shown in Fig. 1a, in the presence of 32 μg/ml FLC, cell growth was significantly inhibited, although single colonies of cells that were likely resistant to FLC were observed. Co-treatment of cells with both FLC and any of the four anti-oxidants led to rescue of growth. While RA, AA and PDTC showed a robust reversal of FLC-mediated growth inhibition, GSH showed only a modest rescue of growth.

It has been previously demonstrated that treatment of *C. neoformans* with FLC causes cytokinesis defects, visible as a multi-budded phenotype [32]. In order to determine whether the antioxidants can reverse the multi-budded phenotype resulting from FLC treatment, we treated *C. neoformans* cells for 16 h with either 32 μg/ml FLC alone or FLC and an antioxidant (either RA at 1 mM, AA at 10 mM, PDTC at 10 μM, or GSH at 10 mM). As shown in Fig. 1b, cells treated with FLC alone exhibited a multi-budded phenotype indicative of a cytokinesis defect, consistent with previous studies (Fig. 1b, arrows). When cells were co-treated with FLC and either AA, RA, PDTC, or GSH, cell morphology was similar to that of the control sample and no significant multi-budded phenotype was observed (Fig. 1b). Thus, the ability of tested antioxidants to rescue *C. neoformans* cells from FLC-mediated growth inhibition correlated with the ability of each of the antioxidants to reduce the multi-budded morphology of cells resulting from FLC treatment. In addition to morphological defects, *C. neoformans* cells exhibited clumping phenotype in the presence of 32 μg/ml FLC. As represented in Fig. 1c, cell aggregates were drastically reduced in the presence of AA, RA, PDTC, or GSH. These findings suggest that the multi-budded phenotype and cell aggregation resulting from FLC treatment is at least partially caused by the increase of ROS, based on the ability of various antioxidants to reverse these phenotypes. Interestingly, while GSH could rescue FLC-induced morphological growth defects, as shown in Fig. 1b, co-treatment of cells with FLC at 32 μg/ml and GSH at 10 mM led to only a modest rescue of growth, in contrast to addition of AA, RA, or PDTC, as shown in Fig. 1a. These findings suggest that the tested anti-oxidants reverse the effects of FLC through mechanisms that may not be identical.

Copper levels are increased during *C. neoformans* infection and the increased copper aids *C. neoformans* virulence, while also contributing to an increase in ROS [25, 30]. Metallothionein proteins (Cmt1 and Cmt2), whose levels increase in response to copper, have been implicated in lowering ROS by sequestering heavy metals such as copper [33]. Since it has been shown that *C. neoformans* mutants lacking metallothioneins are more sensitive to FLC [19], we tested whether metallothioneins are required for antioxidant-mediated growth rescue in the presence of FLC. We carried out a serial dilution assay where we spotted wild type, *cmt1*Δ*, cmt2*Δ*,* or *cmt1/2*Δ mutants as a series of 10-fold diluted cell cultures. As shown in Fig. 2a, all the strains grew equally well on a control YPD medium, while on YPD medium supplemented with FLC (32 μg/ml) all strains were significantly inhibited. However, growth of wild type was robustly rescued when cells were grown on YPD medium supplemented with FLC and RA (Fig. 2a), Growth rescue of the wild type was less evident in the presence of AA, or PDTC, and it was minimal in the presence of GSH. Compared to wild type cells, growth of the metallothionein mutants could not be rescued to the same extent when grown in YPD medium supplemented with FLC and PDTC, AA, RA, or GSH. Consistently, both wild type and *cmt* mutant strains showed maximum rescue from FLC inhibition by RA (Fig. 2a). This finding suggests that metallothionein proteins are necessary for the effective growth rescue by antioxidants when cells are treated with FLC.

To further test whether treatment of wild type vs metallothionein mutant strains (*cmt1*Δ*, cmt2*Δ and *cmt1/2*Δ) results in change in sensitivity to FLC, we carried out a disc diffusion assay, which allowed us to compare the effects of increasing amounts of FLC. We plated 2 × 10^6^ cells of either wild type (H99) or metallothionein mutants on YPD semisolid medium and we placed on the surface of the medium a control disc and 3 discs containing 25, 50, or 100 μg of FLC. As shown in Fig. 2b, all three metallothionein mutant strains exhibited higher sensitivity to FLC, as indicated by larger zones of inhibition surrounding the discs containing FLC, as compared to wild type strain. We also utilized the disc diffusion assays to examine the effects of antioxidants on the sensitivity of metallothionein mutant strains to FLC. As indicated in Fig. 2b, and graphed in Fig. 2c (based on discs containing 100 micrograms of FLC), wild type strain (H99) exhibited a significant recovery from FLC-mediated growth inhibition in the presence of AA, RA, PDTC, and GSH with PDTC appearing as most potent. In contrast, for all three metallothionein mutants, *cmt1*Δ, *cmt2*Δ, or *cmt1/2*Δ, the recovery from FLC mediated inhibition in the presence of PDTC was less significant as compared to the wild type (Fig. 2c). The *cmt1/2Δ* double mutant was unable to significantly recover from FLC-mediated growth inhibition in the presence of AA (Fig. 2b, c). In the presence of RA and GSH, all metallothionein mutants could recover from growth inhibition caused by FLC (Fig. 2b, c). These findings suggest that the antioxidants tested may function in different ways to reverse growth inhibition caused by FLC in *C. neoformans*. PDTC-mediated growth recovery of *C. neoformans* and to a lesser extent also AA-mediated growth recovery of *C. neoformans*, in the presence of FLC, may require expression of metallothioneins, while for RA and GSH to exert their effects metallothioneins activity may not be critical.

Our data suggested that the degree to which the antioxidants reversed the inhibition by FLC was unequal. One explanation of these differences may be the ability of each antioxidant to reduce ROS at applied concentrations. To test this possibility, we examined the capacity of each of the antioxidants to reduce ROS in *C. neoformans*. First, we utilized hydrogen peroxide, which is a well-established ROS-generating agent that induces oxidative stress in *C. neformans* cells, as determined by increased expression of enzymatic antioxidants, including CAT1, CAT3, and TRR1 [34]. We performed a growth spot assay with the wild type (H99) strain in either YPD media plates, YPD supplemented with 3 mM hydrogen peroxide, or YPD supplemented with 3 mM hydrogen peroxide and one of the four antioxidants (AA, RA, PDTC, or GSH). As shown in Fig. 3a, in the presence of 3 mM hydrogen peroxide, growth of cells was dramatically reduced. Strikingly, the presence of 10 mM GSH or 10 mM AA led to a complete rescue of growth from the inhibitory effects of hydrogen peroxide, which indicates that AA and GSH are potent antioxidants in *C. neoformans*. RA and PDTC also rescued growth inhibition by hydrogen peroxide, but not to the same extent as GSH or AA (Fig. 3a). These results were further confirmed by the disc diffusion assay, in which wild type cells were plated on either YPD or YPD media supplemented with an antioxidant (AA, RA, GSH or PDTC) and exposed to a control disc and 3 discs with increasing concentrations of hydrogen peroxide (25, 50, or 100 mM). As shown in Fig. 3b, in the absence of an antioxidant, distinct zones of inhibition were formed that increased in diameter, as the concentration of hydrogen peroxide increased. When YPD was supplemented with the antioxidants GSH and AA, the diameters of the zones of inhibition at all the concentrations of hydrogen peroxide tested were smaller. However, presence of PDTC or RA in the YPD media did not lead to the reduction of the zones of inhibition in the presence of hydrogen peroxide, indicating that AA and GSH were more effective antioxidants to alleviate the effects of hydrogen peroxide in *C. neoformans* when compared to PDTC and RA. Thus, our results suggest that although all the antioxidants could rescue growth inhibition in the presence of hydrogen peroxide, GSH and AA are more potent antioxidants than RA and PDTC in *C. neoformans*.

To further test whether addition of the antioxidants (RA, AA, PDTC, or GSH) could reverse oxidative stress that is present during FLC treatment, we carried out a fluorescent assay to detect Reactive Oxygen Species (ROS). We used a ROS indicator, 4-Amino-5-methylamino-2′,7′-difluorofluorescein diacetate (H_2_DCFDA), which binds to free radicals within the cell and can be detected as a fluorescent signal at an excitation wavelength of 495 nm and emission wavelength of 529 nm, as used in a recent study [19]. As shown in Fig. 3c, treatment with FLC led to elevated ROS and co-treatment with either GSH, AA, RA, or PDTC significantly lowered levels of ROS. While all antioxidants lowered ROS generated in the presence of FLC, GSH and AA led to the most significant decrease in ROS, while the effect of RA and PDTC was less pronounced. Furthermore, GSH and AA appeared to lower ROS significantly below the endogenous levels, while PDTC and RA specifically led to a decrease of ROS generated upon addition of FLC.

Based on data implicating metallothioneins in counteracting the inhibitory effects of FLC, we hypothesized that FLC treatment may lead to a more extensive accumulation of ROS in the metallothionein mutants as compared to the wild type. In order to test whether the ROS generated by wild type (H99) cells was lower compared to metallothionein mutants (either *cmt1*Δ, *cmt2*Δ, or *cmt1/2*Δ), we carried out fluorescence measurements using the dye H_2_DCFDA. As shown in Fig. 3d, the overall ROS was significantly higher in *cmt2*Δ and the *cmt1/2*Δ double mutants as compared to the wild type. These results suggest that the CMT2 metallothionein protein contributes to lowering ROS in *C. neoformans* during FLC treatment.

## Discussion

The main aim of this study was to determine whether treatment with various antioxidants could reverse growth defects and morphological defects caused by FLC treatment in *C. neoformans*. Previous studies have implicated FLC to be involved in generation of ROS in *C. albicans* [16–18] and in *C. neoformans* [19]. It has been shown that AA can rescue growth inhibition caused by FLC in *C. neoformans* [19]. It has been demonstrated that addition of AA leads to induction of *ERG11* gene expression to allow for increased ergosterol production and this process is mediated by a transcription factor Upc2 [23, 24]. Hence, how AA reduces growth inhibition caused by FLC remains unclear. The effect of FLC on ROS in *C. neoformans* shown in the study by Peng et al. appears insignificant compared to the increase of ROS caused by another antifungal drug, amphotericin B [19, 35]. Another azole drug, itraconazole, led to ROS formation and lipid peroxidation in a sibling species *C. gattii* in the early stages of the treatment; this did not occur with fluconazole [36]. Therefore, the contribution of ROS in *C. neoformans* during FLC treatment remains unclear. We reasoned that if antioxidants indeed reverse oxidative stress generated by ROS, then various antioxidants should rescue growth inhibition of *C. neoformans* in the presence of FLC. While we found that all tested antioxidants (AA, RA, PDTC, and GSH) could rescue growth inhibited by FLC (using growth assays, spot assays and disc diffusion assays to assess sensitivity to FLC), the rescue was not uniform. If we consider a measure of growth rescue in the presence of hydrogen peroxide as an indicator of the antioxidant potential, AA and GSH were the most efficient antioxidants in *C. neoformans*, while PDTC and RA were less effective as antioxidants compared to AA and GSH. Interestingly, while GSH was one of the more potent antioxidants, based on the ability of GSH to lower ROS caused by hydrogen peroxide and FLC, GSH only moderately rescued growth inhibition by FLC. This suggests that the antioxidants may be counteracting specific species of ROS not always matching ROS type that is generated during oxidative stress in the presence of FLC. A non-exclusive possibility is that the effects of the antioxidants also involve changes in the expression of ergosterol pathway genes in addition to lowering ROS and collectively contribute to the survival in the presence of FLC. Future studies that would examine ergosterol levels and measure global gene expression in the presence of FLC and specific antioxidants will help to resolve these uncertainties.

Interestingly, each of the antioxidants tested could rescue morphological growth defects caused by FLC. *C. neoformans* wild type (H99) strain treated with (FLC) at 32 μg/mL displayed multi-budded phenotype most likely due to inability to perform cytokinesis. Our study revealed that all the antioxidants tested (AA, RA, PDTC, and GSH) can rescue cells from cytokinesis defects caused by FLC, but not all antioxidants could rescue growth inhibition due to FLC to the same extent. This result suggests that while cytokinesis defect may contribute to growth defect in FLC-treated cells, eliminating this aberration is not sufficient to restore growth in the presence of FLC.

Another evidence that FLC is contributing to an increase in ROS in *C. neoformans* is the involvement of the metallothionein genes *CMT1* and *CMT2* in resisting the inhibitory effects of FLC. Metallothionein proteins are essential for sequestering copper levels, which are upregulated during infection by *C. neoformans* [29]. Increased copper levels can induce ROS, hence during infection by *C. neoformans*, expression of CMT genes is crucial. Previous studies have shown that mutants of *C. neoformans*, lacking *cmt* genes are more sensitive to FLC treatment [19]. Our results suggest that cells lacking Cmt2 or both Cmt1 and Cmt2 proteins are more sensitive to FLC. We also find that Cmt mutants are compromised in their ability to recover *C. neoformans* cells from FLC treatment when antioxidants are added. Interestingly, the extent to which the mutants could be rescued when co-treated with FLC and an antioxidant varied depending on the type of antioxidant. We find that Cmt proteins play an important role in PDTC-based growth rescue in FLC-treated cells. This further suggests that these antioxidants act through various molecular mechanisms to facilitate rescue from FLC-mediated cell growth inhibition. Future studies should determine the effects of the antioxidants on gene expression in cells treated with FLC. Including Cmt mutants in transcriptional profiling of *C. neoformans* during various treatments would shed light on molecular mechanisms responsible for FLC resistance in *C. neoformans*.

The antioxidants we have tested in this study have been shown to reverse damage caused by many types of free radicals. PDTC has been shown to reverse oxidative damage and carbonylation of proteins by reversing HOCl-mediated oxidative stress [37]. RA has been implicated in hydroxyl radical and lipid peroxide scavenging [38]. AA has been shown to reverse oxidative stress mainly caused by oxygen free radicals [39, 40]. GSH has been implied in reversing oxidative stress generated by hydrogen peroxide [41] and lipid peroxides [42]. Previous studies using *C. glabrata* as a model have suggested that FLC causes an increase in singlet oxygen and peroxide radicals and can cause DNA damage and treatment of *Candida* with FLC increased activity of enzymatic antioxidants, namely superoxide dismutase (SOD) and glutathione peroxidase (GPx) [16]. It is possible that reversal of ROS and growth defects in the presence of AA and GSH in *C. neoformans* is due to the quenching of singlet oxygen species and hydrogen peroxide damage caused by FLC. In addition to DNA damage, it is possible that protein oxidation and carbonylation could be increased in the presence of FLC, which would explain the role played by PDTC in reversing FLC damage in *C. neoformans.* FLC has been shown to be more potent in *Candida* species strains defective in superoxide dismutase and catalase activity [43]. Hence RA could have restored FLC-mediated growth inhibition by regulated SOD levels in the presence of FLC. Further investigations should determine what specific forms of free radicals are upregulated in the presence of FLC and the extent of DNA and protein damage that could be caused in the presence of FLC.

## Conclusions

In summary, we conclude that one of the effects of FLC treatment in *C. neoformans* is an increase in ROS. Furthermore, addition of antioxidants can partially rescue growth of *C. neoformans* in the presence of FLC. However, our results point to a complex nature of the effects of the antioxidants and suggest that various mechanisms contribute to the antioxidant-mediated growth rescue. The significance of this study is in understanding environmental conditions that can cause rescue of growth of *C. neoformans* in the presence of FLC and potentially development of resistance to FLC. While formation of aneuploid cells is associated with FLC resistance, recent studies are revealing that counteracting ROS caused by FLC in fungi could also contribute to resisting FLC mode of action. Understanding how individual antioxidants could reverse ROS generated by FLC and tying their effects to transcriptional profiling of genes that get altered during co-treatment with FLC and antioxidants would uncover molecular mechanisms that potentially lead to FLC resistance in *C. neoformans* and other pathogenic fungi.

## Methods

### Reagents used

Ascorbic Acid or AA (Fisher Scientific, Cat No A61-25, CAS 5081-7) was prepared from a stock of 1 M, and used at 10 mM. A reduced form of glutathione or GSH (Alfa Aesar, Cat No AAJ6216606, CAS 70-18-8) was prepared from a stock of 0.5 M, and used at 10 mM. Pyrrolidinedithiocarbamate or PDTC (Cayman Chemicals, Cat No 20713, CAS 5108-96-3) was prepared from a stock of 10 mM, and used at 10 μM. Retinoic Acid or RA (Cayman Chemical, Cat No 11017, CAS 302-79-4) was prepared from a stock of 100 mM (dissolved in dimethyl sulfoxide (DMSO)), and used at 1 mM. The fluorescent dye for ROS assays, 4-Amino-5-methylamino-2′,7′-difluorofluorescein diacetate (H_2_DCFDA) (Sigma, Cat No D6883, CAS 4091-99-0), was dissolved in DMSO at a stock concentration of 100 mM and used at 10 μM. Fluconazole (Cayman Chemical, Cat No 11594, CAS 86386-73-4) was dissolved in DMSO as a 50 mg/ml stock and used at 32 μg/ml. Hydrogen peroxide (Cat No H325-100) was obtained from Fisher Scientific.

### Strains and media

*Cryptococcus neoformans* var. *grubii* wild type (strain H99 Stud) is the derivative of the original strain isolated in 1978 by John Perfect at Duke University (ATCC 208821) that has been passaged through a rabbit at that time. The *cmt1*Δ, *cmt2*Δ, *cmt1/2*Δ deletion mutants isogenic to H99 (*CMT1*, CNAG_05549; *CMT2*, CNAG_00306) were kindly provided by the laboratory of Dr. Lukasz Kozubowski, Clemson University (the metallothionein mutants were originally obtained from Dr. Dennis J. Thiele, Duke University).

Cells were grown on YPD media: (1% yeast extract, 2% peptone, 2% dextrose, 2% agar), supplemented with chemicals as indicated in the text.

### Fluconazole sensitivity plate and spot growth assays

Either wild type, *cmt1*Δ, *cmt2*Δ or *cmt1/2*Δ were grown in liquid YPD broth overnight for 16 h. All strains were diluted to an Optical Density of OD_600_ = 0.1 and refreshed in YPD liquid media for 4 h and then counted using a Neubauer Hemocytometer. For growth assays, ~ 10,000 cells in exponential growth phase were spread onto plates containing either YPD media alone, YPD plus 32 μg/μL FLC, and YPD plus 32 μg/μL FLC and an antioxidant, namely, 10 μM PDTC, 1 mM RA, 10 mM AA, or 10 mM GSH. Spot growth assays were performed with a 10-fold serial dilution of cells such that 2 μL contained either 10^4^, 10^3^, 10^2^, or 10 cells and were carefully spotted onto YPD plates alone, YPD plus 32 μg/μL FLC, or YPD plus FLC and individual antioxidants, as described above. For both growth assays and spot assays, cells grew for 48 h at 25 °C before recording the data.

### Fluorescence assay to detect ROS

Cells were grown overnight at room temperature in 2 ml liquid YPD medium with constant agitation, diluted to an Optical Density OD_600_ = 0.1, and grown for an additional 4 h. Subsequently, the culture was diluted to 10,000 cells/ml and the cultures were either grown as no treatment control, treated with either 32 μg/ml FLC, or 32 μg/ml FLC and an antioxidant (either 10 μM PDTC, 1 mM, RA, 10 mM AA, or 10 mM GSH) for 12 h. To detect ROS, 10 μM of a fluorescent dye, H_2_DCFDA, was added to each of the samples and incubated for 1 h in the dark at 25 °C. A control set of each of the samples were incubated without the fluorescent dye. 250 μL of the sample were added to each well of a 96-well microplate. ROS was measured as fluorescence emitted by the fluorescent dye, H_2_DCFDA, at an excitation wavelength of 485 nm and an emission wavelength of 535 nm. The fluorescence reading was measured and recorded as Relative Fluorescence Units (RFU). From each reading of the sample treated with H_2_DCFDA the reading obtained from the sample without addition of H_2_DCFDA was subtracted. Each treatment was made in triplicate. All data points were computed using multifactorial ANOVA and Tukey’s HSD post hoc test.

### Disk diffusion assay

*C. neoformans* strains (wild type H99, or mutants, *cmt1*Δ*, cmt2*Δ*, or cmt1/2*Δ) were grown in 2 ml of YPD liquid broth overnight, for 16 h, diluted to an OD_600_ = 0.1 and refreshed for 4 h. Each strain was counted using a hemocytometer and ~ 2 × 10^6^ cells were plated onto YPD semi-solid media plates containing either no antioxidant (control), AA (10 mM), RA (1 mM), PDTC (10 μM) or GSH (10 mM), and spread with sterile Dynarex cotton tipped applicators at opposing 90° angles. The plates were left to dry before application of cotton disks. After 10 min of drying, 6.6 mm cotton disks were lightly placed perpendicular on top of the YPD medium as to not break the surface of the gel. Depending on the experiment, either increasing amounts of 25, 50 and 100 micrograms of FLC, or increasing concentrations of 25, 50, and 100 mM hydrogen peroxide were added to the top end of the disk in order for the FLC or hydrogen peroxide to diffuse throughout the area surrounding the disc. Finally, the discs were laid flush onto the medium equidistant from one another. The cells grew for 48 h at 25 °C and all treatments were done in triplicate. Each zone of inhibition was measured and the results from each of the three replicate experiments were averaged. A multifactorial ANOVA along with a Tukey’s HSD post hoc test was used to indicate significance.

### Microscopy

Differential interference contrast (DIC) microscopy was used to study *C. neoformans* cell morphology under various conditions. *C. neoformans* cells were grown for 16 h at 25 °C in YPD liquid media, diluted down to an OD_600_ = 0.1, and refreshed for 4 h. Cells were then grown with either no treatment (control cells), treatment with FLC alone at 32 μg/ml, or FLC at 32 μg/ml and an antioxidant (10 μM PDTC, 1 mM RA, 10 mM AA, or 10 mM GSH) for 16 h. Cells were centrifuged at 3000 x g for 2 min and washed with ice cold PBS (137 mM NaCl, 2.7 mM KCl, 10 mM Na_2_HPO_4_, 1.8 mM KH_2_PO_4_). An agar trap was made to capture yeast cells, by melting 0.8% agarose on a slide as a thin section. Cells were placed in an agar trap, covered with a coverslip and visualized by Zeiss Axiovert 200 inverted microscope (Carl zeiss, Inc., Thornwood, NY).

### Statistical analyses

For all statistical analyses, the Shapiro Wilk Test was used to test for normality, and afterward, the Bartlett Test was used to test for equality of variance. Since both conditions were met, a multifactorial ANOVA was performed. The Tukey HSD test was used to determine if the relationship between the control group and variable groups were statistically significant.

## Supplementary information

**Additional file 1.** Raw data corresponding to Figures 1C, 2C, 3C, and 3D that are included in the article.

## Data Availability

All data generated during this study are included in this pulished article and in Additional file 1, which contains raw data corresponding to Figures 1C, 2C, 3C, and 3D.

## Abbreviations

AA: Ascorbic acid
FLC: Fluconazole
GSH: Glutathione
H_2_DCFDA: 4-Amino-5-methylamino-2′,7′-difluorofluorescein diacetate
MIC: Minimum inhibitory concentration
PDTC: Pyrrolidine dithiocarbamate
RA: Retinoic acid
ROS: Reactive oxygen species

## References

[1] Brown SP, Cornforth DM, Mideo N (2012). Evolution of virulence in opportunistic pathogens: generalism, plasticity, and control. Trends Microbiol.

[2] Idnurm A (2005). Deciphering the model pathogenic fungus Cryptococcus neoformans. Nat Rev Microbiol.

[3] Park BJ (2009). Estimation of the current global burden of cryptococcal meningitis among persons living with HIV/AIDS. Aids.

[4] Assing K, Birgens H, Arendrup M (2003). Cryptococcus neoformans var neoformans resistant to fluconazole in an HIV-negative patient with chronic lymphocytic leukemia. Clin Microbiol Infect.

[5] Chen YC (2015). Increasing trend of fluconazole-non-susceptible Cryptococcus neoformans in patients with invasive cryptococcosis: a 12-year longitudinal study. BMC Infect Dis.

[6] Smith KD (2015). Increased antifungal drug resistance in clinical isolates of Cryptococcus neoformans in Uganda. Antimicrob Agents Chemother.

[7] Pasko MT, Piscitelli SC, Van Slooten AD (1990). Fluconazole: a new triazole antifungal agent. DICP.

[8] Goa KL, Barradell LB (1995). Fluconazole. An update of its pharmacodynamic and pharmacokinetic properties and therapeutic use in major superficial and systemic mycoses in immunocompromised patients. Drugs.

[9] Li Z (2019). Fluconazole plus flucytosine is a good alternative therapy for non-HIV and non-transplant-associated cryptococcal meningitis: a retrospective cohort study. Mycoses.

[10] Khan AA (2018). Additive potential of combination therapy against cryptococcosis employing a novel amphotericin B and fluconazole loaded dual delivery system. Eur J Pharm Sci.

[11] Revankar SG (2004). Cloning and characterization of the lanosterol 14alpha-demethylase (ERG11) gene in Cryptococcus neoformans. Biochem Biophys Res Commun.

[12] Sionov E (2010). Cryptococcus neoformans overcomes stress of azole drugs by formation of disomy in specific multiple chromosomes. PLoS Pathog.

[13] Gast CE (2013). Azole resistance in Cryptococcus gattii from the Pacific northwest: investigation of the role of ERG11. Antimicrob Agents Chemother.

[14] Posteraro B (2003). Identification and characterization of a Cryptococcus neoformans ATP binding cassette (ABC) transporter-encoding gene, CnAFR1, involved in the resistance to fluconazole. Mol Microbiol.

[15] Cowen LE (2014). Mechanisms of antifungal drug resistance. Cold Spring Harb Perspect Med.

[16] Mahl CD (2015). Induction of ROS generation by fluconazole in Candida glabrata: activation of antioxidant enzymes and oxidative DNA damage. Diagn Microbiol Infect Dis.

[17] Wang Y (2009). Ascorbic acid decreases the antifungal effect of fluconazole in the treatment of candidiasis. Clin Exp Pharmacol Physiol.

[18] Kobayashi D (2002). Endogenous reactive oxygen species is an important mediator of miconazole antifungal effect. Antimicrob Agents Chemother.

[19] Peng CA (2018). Fluconazole induces ROS in Cryptococcus neoformans and contributes to DNA damage in vitro. PLoS One.

[20] Winterbourn CC (2008). Reconciling the chemistry and biology of reactive oxygen species. Nat Chem Biol.

[21] Angele-Martinez C, Goodman C, Brumaghim J (2014). Metal-mediated DNA damage and cell death: mechanisms, detection methods, and cellular consequences. Metallomics.

[22] Fedorova M, Bollineni RC, Hoffmann R (2014). Protein carbonylation as a major hallmark of oxidative damage: update of analytical strategies. Mass Spectrom Rev.

[23] Van Hauwenhuyse F, Fiori A, Van Dijck P (2014). Ascorbic acid inhibition of Candida albicans Hsp90-mediated morphogenesis occurs via the transcriptional regulator Upc2. Eukaryot Cell.

[24] Yang H (2015). Structural mechanism of ergosterol regulation by fungal sterol transcription factor Upc2. Nat Commun.

[25] Raja MR (2013). A copper hyperaccumulation phenotype correlates with pathogenesis in Cryptococcus neoformans. Metallomics.

[26] Zhang P (2016). Effects of CTR4 deletion on virulence and stress response in Cryptococcus neoformans. Antonie Van Leeuwenhoek.

[27] Jiang N (2009). A copper-responsive factor gene CUF1 is required for copper induction of laccase in Cryptococcus neoformans. FEMS Microbiol Lett.

[28] Husain N, Mahmood R (2019). Copper (II) generates ROS and RNS, impairs antioxidant system and damages membrane and DNA in human blood cells. Environ Sci Pollut Res Int.

[29] Ding C (2011). The copper regulon of the human fungal pathogen Cryptococcus neoformans H99. Mol Microbiol.

[30] Ding C (2013). Cryptococcus neoformans copper detoxification machinery is critical for fungal virulence. Cell Host Microbe.

[31] Niedzwiecka K (2016). Glutathione may have implications in the design of 3-bromopyruvate treatment protocols for both fungal and algal infections as well as multiple myeloma. Oncotarget.

[32] Altamirano S, et al. Fluconazole-induced ploidy change in Cryptococcus neoformans results from the uncoupling of cell growth and nuclear division. mSphere. 2017;2(3):e00205-17. 10.1128/mSphere.00205-1. 10.1128/mSphere.00205-17 PMC5471349 28630940

[33] Vasak M (2005). Advances in metallothionein structure and functions. J Trace Elem Med Biol.

[34] Upadhya R (2013). Global transcriptome profile of Cryptococcus neoformans during exposure to hydrogen peroxide induced oxidative stress. PLoS One.

[35] Mesa-Arango AC (2014). The production of reactive oxygen species is a universal action mechanism of amphotericin B against pathogenic yeasts and contributes to the fungicidal effect of this drug. Antimicrob Agents Chemother.

[36] Ferreira GF (2013). The role of oxidative and nitrosative bursts caused by azoles and amphotericin B against the fungal pathogen Cryptococcus gattii. J Antimicrob Chemother.

[37] Zhu BZ, Carr AC, Frei B (2002). Pyrrolidine dithiocarbamate is a potent antioxidant against hypochlorous acid-induced protein damage. FEBS Lett.

[38] Ahlemeyer B (2001). Retinoic acid reduces apoptosis and oxidative stress by preservation of SOD protein level. Free Radic Biol Med.

[39] Zyracka E (2005). Ascorbate abolishes auxotrophy caused by the lack of superoxide dismutase in Saccharomyces cerevisiae. Yeast can be a biosensor for antioxidants. J Biotechnol.

[40] Boatright WL (2016). Oxygen dependency of one-electron reactions generating ascorbate radicals and hydrogen peroxide from ascorbic acid. Food Chem.

[41] Munro D, Treberg JR (2017). A radical shift in perspective: mitochondria as regulators of reactive oxygen species. J Exp Biol.

[42] Conrad M (2018). Regulation of lipid peroxidation and ferroptosis in diverse species. Genes Dev.

[43] Linares CE (2013). Fluconazole and amphotericin-B resistance are associated with increased catalase and superoxide dismutase activity in Candida albicans and Candida dubliniensis. Rev Soc Bras Med Trop.

